# Effect of wearing medical protective masks on treadmill running performance in the postpandemic era: a randomised trial

**DOI:** 10.1186/s13102-022-00598-9

**Published:** 2023-01-11

**Authors:** I-Lin Wang, Yu Su, Shun Yao, Yu-Hong Jiang, Hao-Yu Li, Chien-Ying Lai

**Affiliations:** 1 College of Physical Education, Hubei Normal University, Huangshi, 435000 Hubei China; 2 Graduate Institute, Jilin Sport University, Changchun, 130022 Jilin China; 3 Graduate Institute, Hubei Normal University, Huangshi, 435000 Hubei China; 4 Orthopedic Department, China Medical University Hospital, Taichung, 40447 Taiwan

**Keywords:** COVID-19, Sports performance, Injury, Outdoor or gym sport, Exercise duration

## Abstract

**Background:**

In the postpandemic era, wearing protective masks in public places will still be an important means of blocking popular viruses in the future. The purpose of this study was to explore whether sports performance was affected by mask wearing and exercise duration during 15-min treadmill running at a speed of 75% maximal aerobic speed.

**Methods:**

Thirty-six males were randomly divided into mask and nonmask groups. The kinematic and kinetic data were obtained at four time points (RN_0–1 min_, RN_5–6 min_, RN_9–10 min_, and RN_14–15 min_) during running. Two-way mixed ANOVA was applied to examine the effects between groups and times with Bonferroni post hoc comparison and independent samples t-test.

**Results:**

The results showed that there was no difference between mask and nonmask group during running (p > 0.05). As running time increased, hip joint ROM, hip joint flexion/extension max, and ankle joint plantarflexion max angles increased; knee joint flexion min and ankle joint dorsiflexion max angles decreased; average peak vertical ground reaction forces (PVGRF) increased after 9 min-running (p < 0.05).

**Conclusions:**

Wearing a medical protective mask does not affect the joint angle and touchdown PVGRF of lower extremities during treadmill running while affected by running time and changed after 9 min-treadmill running. Future studies will examine the effects of wearing masks during the pandemic on muscle activation and blood biochemical values during exercise.

*Trial registration* No. ChiCTR2000040535 (date of registration on December 1, 2020). Prospectively registered in the Chinese Clinical Trial Registry.

## Introduction

In March 2020, the World Health Organization (WHO) announced that COVID-19 was a pandemic disease caused by novel coronaviruses (SARS-COV-2). Coping strategies during the COVID-19 pandemic mandated the use of medical protective masks for outdoor and indoor sports activities, but there is still much debate about whether wearing masks during exercise triggers safety issues [1]. To date, during the outbreak, the possible droplet and contact transmission of COVID-19 in public places and possible aerosol transmission within 3–6 feet have been reduced to some extent by wearing protective medical masks and maintaining a safe distance [2]. Many government authorities began to allow the gradual reopening of indoor sports centers, such as gyms, natatoriums, badminton halls, and billiards halls, in May–June 2020, but indoor exercise required wearing masks to ensure safety during the pandemic [3]. Therefore, especially facing the new COVID-19 variants (delta, omicron, deltacron), outdoor and indoor activities still require mask wearing to actively comply with government regulations and prevent possible risks of future pandemics.

Wearing a mask increases airflow resistance and prevents the body from receiving the required air at the maximum rate [4]. Past studies have found that wearing a cloth face mask while running on a treadmill significantly reduced overall exercise time by 14% and maximal aerobic capacity (VO_2_ max) by 29% compared with nonmask, and higher levels of exercise can also cause discomfort reactions such as shortness of breath and claustrophobia [5]. Wearing a surgical mask while running caused a decrease in blood oxygen saturation, which reduced the anaerobic capacity of running and increased the metabolic burden on the body's cardiovascular system and other organs [1]. However, another study found that wearing nondisposable cloth masks or disposable surgical masks had no direct impact on sports performance during moderate intensity exercise or vigorous exercise [6]. Currently, studies on exercise with masks have not defined the exercise conditions that each participant can adapt to his or her own ability based on VO_2_ max. Therefore, whether wearing medical protective masks during exercise affects sports performance and increases the risk of injury still needs to be explored by standardizing VO_2_ max.

The biomechanics of running, such as the foot initial angle with contact surface, plantar pressure distribution, step length, acceleration, and vertical ground reaction force (VGRF), are adaptively altered by the fatigue state of the body [7]. Specifically, VGRF is often used to investigate the risk of lower extremity injury and assess performance during running. Past studies found that when running on a treadmill at a speed of 2.9 m/s, muscle fatigue reduces the ability of the lower extremities to cushion impact forces from the contact surface, thereby increasing VGRF and loading rate of the impact force [8, 9]. However, other studies have found that fatigue caused by running on a treadmill at a speed of 2.7 ~ 4.5 m/s reduces the impact peaks and loading rates of the lower extremities [10]. Therefore, the influence of running-induced fatigue on VGRF at touchdown should be further demonstrated by considering the runner's speed. Moreover, past studies have concluded that the good facial fit and filtration properties of medical surgical masks can impede human breathing and heat dissipation to some extent [11]. The extra respiratory muscle work may make the body more prone to fatigue, leading to higher impact and even a higher risk of lower extremity injuries during running. Therefore, it is necessary to further explore the influence of respiratory resistance on body fatigue when wearing a medical mask under the condition of adequate protection.

In the postpandemic era, it is still mandatory for most countries and regions to wear masks when exercising in gyms; due to safety considerations, most people still choose to wear masks for exercise. Whether wearing medical protective masks to prevent respiratory droplets or aerosol particles carrying COVID-19 virus from passing through will affect running performance by causing discomfort symptoms such as dyspnea requires further discussion to provide reference results. The purpose of this study was to explore whether lower extremity sports performance was affected by mask wearing and exercise duration during 15-min treadmill running at a speed of 75% VO_2_ max. In this study, we hypothesized that medical mask wearing and running time would affect lower extremity performance and increase injury risk.

## Materials and methods

### Participants

Thirty-six healthy males from Jilin Sport University (age: 20 ± 1 years; body mass: 71.1 ± 10.0 kg; and height: 177 ± 6.4 cm) were recruited one month prior to participate in this study without a history of lower extremity musculoskeletal pathology, neurological, or cardiopulmonary diseases that would affect running gait to maintain balance [12]. They were randomly divided into a mask group (wearing a medical protective mask, n = 18) and a nonmask group (without a medical protective mask, n = 18) with body mass index (BMI) ranging from 18.5 to 24 kg/m^2^. All experimental procedures followed the principles of the Helsinki Declaration.

### Experimental design and data collection

An exhaustive treadmill running test (Intertrack 8100, Schiller AG, Switzerland) was carried out via a face mask using a cardiopulmonary ergospirometry instrument (Schiller CS200, Schiller AG, Switzerland). Combined with height and weight anthropometric measurements, the steady-state submaximal exercise test was conducted in standard laboratory conditions with ambient temperature and relative humidity of 20 °C and 50%, respectively. Three days before the start of the experiment, participants were asked to abstain from any strenuous exercise that would affect the test. The VO_2_ max measurement was conducted on the motor driven treadmill using the ramp incremental protocol. The initial speed on the treadmill was 6 m/s and was then increase 1 m/s every 6-min until occurring risk factors or volitional exhaustion. The linear relationship between running speed and maximal aerobic capacity was subsequently used to calculate workload for the running trials, as 75% VO_2_ max [13]. The exercise intensity of tandem treadmill (Force-sensing tandem treadmill, AMTI, USA) running experiment was based on 75% individual’s VO_2_ max.

The three-dimensional optical motion analysis system included a 10-camera (Vicon V5 cameras, Vicon Motion Systems® Ltd., Oxford, UK) and Nexus software (Version 2.9.0, Vicon Motion Systems®, UK) sampling at 200 Hz to capture marker trajectory kinematic data. The force-sensing treadmill was equipped with two tandem force plates below treadmill belts mounted in anteroposterior tandem with clearance less than 10 mm, which collected kinetic data on the front force plate of the treadmill at 2000 Hz and synchronized with the motion capture system. Using a low-pass fourth-order Butterworth filter to smooth marker trajectories and force plate data with cut-off frequencies of 6 and 20 Hz [14]. We normalized all ground reaction forces with respect to body weight (BW). During the running stance phase, kinetic data were analyzed and defined as the interval from heel strike (vertical GRF greater than 20 N) to toe off (vertical GRF less than 20 N). Plug-in Gait model was used to identify the 7-segment rigid link model of the lower extremities. Marker trajectories and kinetic data were labelled and exported as C3D files and uploaded into MATLAB (version R2019a; MathWorks, Inc., Natick, MA).

### Testing procedures

**Fig. 1:**
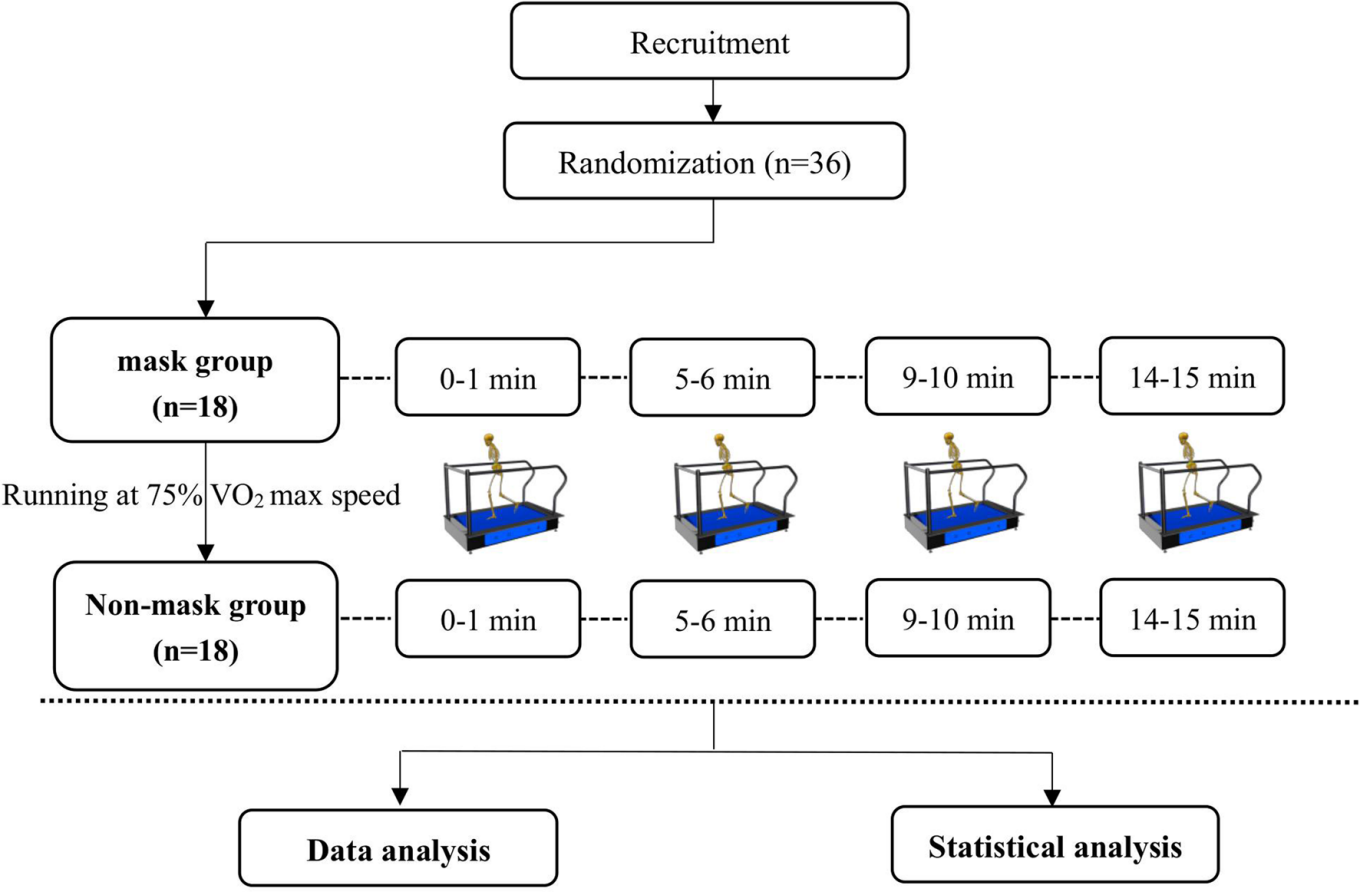
Study flow diagram

Anthropometric measurements were taken, and 20 retroreflective markers were placed on the participants’ lower extremity anatomical landmarks following the modified Plug-In-Gait model in Vicon Nexus software. Participants were required to complete a 15-min warm-up freely on a treadmill at 75% of individual VO_2_ max speed until they were familiar with all experimental procedures. Following 10 min of rest, each participant wore shorts and shoes uniformly provided by the laboratory and was asked to run on the front force plate of the treadmill at 75% of the individual VO_2_ max speed for 15 min. The kinematic and kinetic data were obtained at four time points (RN_0–1 min_, RN_5–6 min_, RN_9–10 min_, and RN_14–15 min_) during running (Fig. 1).

### Data analysis

The kinematic and kinetic variables of the hip, knee, and ankle joints in the sagittal planes for each stance phase during 15 min of running included the following: (1) hip joint: average maximum flexion/extension angles (flexion max, extension max); (2) knee joint: average maximum flexion angle (flexion max), average minimum flexion angle (flexion min); and (3) average maximum dorsiflexion/plantar flexion angles (dorsiflexion max, plantarflexion max); (4) average joint ranges of motion (ROM) (ROM = flexion max-extension max/flexion max-flexion min/dorsiflexion max-plantarflexion max); (5) average peak vertical ground reaction force (PVGRF) for each stance phase. Average PVGRFs were analyzed using MATLAB. Functional definitions of the hip, knee, ankle joint centers, and axes were determined by a modified Plug-In-Gait model [15, 16]. Lower extremity angles at the vertical position anteriorly were defined as positive, and the vertical position posteriorly was defined as negative.

### Statistical analysis

All statistical analyses were performed in MATLAB (Version 2019a, MathWorks Inc., Natick, MA). A two-way mixed model ANOVA was applied to examine the effects between group (mask, nonmask) and time (RN_0–1 min_, RN_5–6 min_, RN_9–10 min_ and RN_14–15 min_). Comparisons of two groups at every time point were performed after testing for normality by using the Shapiro–Wilk test [17]. The unpaired t tests of groups and one-way repeated measures ANOVA of times by Bonferroni post hoc comparison method were performed with the significance level set to p < 0.05. Cohen’s d effect size (ES: “small” around 0.2, “medium” about 0.5, “large” greater than 0.8 [18]) were computed to illustrate differences between conditions. Furthermore, a sample size estimation was conducted following a priori power analysis (G*Power version 3.1.9.7; Heinrich Heine University Düsseldorf, Germany) based on conventional α err prob (0.05), power (1-β err prob) (0.80) values and an effect size of 0.82, subsequently, a recommended minimum total sample size of 33 participants was found.

## Results

No significant interaction was found (Table 1 and Table 2) between the two factors (Group *Times) for analyzing any dependent variable (p > 0.05). The effect of the lower extremity average joint angle and average PVGRF change over time during running on the study dependent variable was not modified by whether the runner wore a mask. Therefore, differences in the main effect within times were detected, and the lower extremity average joint angle and average PVGRF changed after nine minutes of running. However, the main effects of the groups did not reach significant differences.

**Table 1:** Kinematic variables for joint motion during treadmill running between the mask and nonmask groups

Characteristic	RN_time	RN_nonmask	RN_mask	P values		
				Main effect (Group)	Main effect (Times)	Interaction (Group × Times)
Right Hip Average Flexion Max (°)	0–1 min	25.808 ± 5.633	26.543 ± 5.390	0.905	0.002*	0.364
	5–6 min	27.569 ± 6.656	26.855 ± 4.332			
	9–10 min	27.859 ± 6.330	27.509 ± 4.973			
	14–15 min	28.278 ± 6.723	27.734 ± 4.936			
Left Hip Average Flexion Max (°)	0–1 min	26.594 ± 6.163	27.181 ± 4.884	0.742	0.454	0.713
	5–6 min	26.870 ± 5.986	27.542 ± 4.309			
	9–10 min	26.917 ± 5.959	27.772 ± 4.607			
	14–15 min	27.196 ± 6.148	27.412 ± 4.791			
Right Hip Average Extension Max (°)	0–1 min	− 10.682 ± 6.204	− 10.814 ± 5.386	0.717	0.008*	0.291
	5–6 min	− 10.639 ± 5.202	− 11.986 ± 5.345			
	9–10 min	− 11.476 ± 6.503	− 12.083 ± 5.279			
	14–15 min	− 11.565 ± 6.079	− 12.190 ± 5.284			
Left Hip Average Extension Max (°)	0–1 min	− 11.331 ± 5.260	− 11.376 ± 4.925	0.879	0.009*	0.163
	5–6 min	− 11.402 ± 5.227	− 12.409 ± 4.498			
	9–10 min	− 12.082 ± 5.953	− 12.721 ± 4.504			
	14–15 min	− 13.094 ± 5.699	− 12.402 ± 4.416			
Right Hip Average ROM (°)	0–1 min	36.489 ± 3.923	37.357 ± 6.426	0.793	0.000*	0.856
	5–6 min	38.208 ± 4.422	38.841 ± 6.593			
	9–10 min	39.335 ± 4.374	39.592 ± 6.519			
	14–15 min	39.844 ± 5.109	39.924 ± 6.630			
Left Hip Average ROM (°)	0–1 min	37.925 ± 4.871	38.557 ± 6.078	0.636	0.009*	0.167
	5–6 min	38.273 ± 4.704	39.951 ± 5.850			
	9–10 min	39.000 ± 4.450	40.493 ± 6.267			
	14–15 min	40.291 ± 5.628	39.814 ± 6.319			
Right Knee Average Flexion Max (°)	0–1 min	61.887 ± 14.775	65.117 ± 14.145	0.510	0.460	0.721
	5–6 min	60.621 ± 14.207	62.844 ± 12.690			
	9–10 min	59.725 ± 11.722	64.095 ± 13.576			
	14–15 min	62.719 ± 15.764	64.092 ± 12.267			
Left Knee Average Flexion Max (°)	0–1 min	59.320 ± 13.383	63.313 ± 12.619	0.305	0.248	0.885
	5–6 min	56.957 ± 11.254	60.181 ± 9.166			
	9–10 min	57.232 ± 10.890	61.807 ± 11.954			
	14–15 min	58.593 ± 12.270	61.654 ± 12.516			
Right Knee Average Flexion Min (°)	0–1 min	9.966 ± 3.746	12.008 ± 3.950	0.350	0.001*	0.163
	5–6 min	9.703 ± 3.814	10.583 ± 3.822			
	9–10 min	9.135 ± 4.126	10.241 ± 3.581			
	14–15 min	9.265 ± 4.024	9.890 ± 3.764			
Left Knee Average Flexion Min (°)	0–1 min	8.884 ± 4.742	10.547 ± 4.040	0.436	0.001*	0.182
	5–6 min	8.433 ± 4.800	9.666 ± 3.651			
	9–10 min	8.193 ± 4.925	9.363 ± 3.881			
	14–15 min	8.264 ± 5.174	8.765 ± 4.075			
Right Knee Average ROM (°)	0–1 min	51.921 ± 13.569	53.110 ± 13.017	0.680	0.441	0.814
	5–6 min	50.919 ± 13.591	52.261 ± 11.678			
	9–10 min	50.591 ± 10.873	53.853 ± 13.118			
	14–15 min	53.453 ± 15.142	54.202 ± 11.451			
Left Knee Average ROM (°)	0–1 min	50.436 ± 11.583	52.765 ± 10.914	0.448	0.323	0.911
	5–6 min	48.524 ± 9.471	50.515 ± 10.127			
	9–10 min	49.039 ± 8.712	52.445 ± 13.225			
	14–15 min	50.329 ± 10.613	52.889 ± 13.406			
Right Ankle Average Dorsiflexion Max (°)	0–1 min	16.811 ± 2.082	15.273 ± 3.084	0.514	0.002*	0.287
	5–6 min	15.610 ± 4.115	14.571 ± 3.455			
	9–10 min	14.119 ± 3.815	13.845 ± 3.307			
	14–15 min	13.291 ± 3.653	13.924 ± 2.717			
Left Ankle Average Dorsiflexion Max (°)	0–1 min	16.536 ± 2.792	16.300 ± 2.874	0.845	0.002*	0.714
	5–6 min	15.499 ± 3.328	15.626 ± 3.348			
	9–10 min	14.830 ± 3.214	15.216 ± 3.159			
	14–15 min	14.771 ± 3.161	15.232 ± 3.062			
Right Ankle Average Plantarflexion Max (°)	0–1 min	− 14.687 ± 5.440	− 16.031 ± 6.342	0.883	0.004*	0.231
	5–6 min	− 17.323 ± 7.163	− 16.557 ± 7.422			
	9–10 min	− 17.522 ± 6.758	− 17.183 ± 6.698			
	14–15 min	− 17.000 ± 5.879	− 17.973 ± 6.229			
Left Ankle Average Plantarflexion Max (°)	0–1 min	− 17.200 ± 8.069	− 15.427 ± 6.897	0.563	0.170	0.561
	5–6 min	− 17.768 ± 6.231	− 17.303 ± 7.198			
	9–10 min	− 18.214 ± 6.274	− 16.683 ± 8.210			
	14–15 min	− 17.999 ± 6.579	− 16.299 ± 8.751			
Right Ankle Average ROM (°)	0–1 min	31.498 ± 6.469	31.304 ± 6.164	0.902	0.780	0.311
	5–6 min	32.933 ± 8.221	31.128 ± 8.131			
	9–10 min	31.640 ± 8.491	31.028 ± 7.891			
	14–15 min	30.689 ± 8.235	32.150 ± 7.199			
Left Ankle Average ROM (°)	0–1 min	33.736 ± 7.713	31.727 ± 7.334	0.638	0.529	0.623
	5–6 min	33.267 ± 6.522	32.929 ± 8.270			
	9–10 min	33.044 ± 6.639	31.900 ± 9.476			
	14–15 min	32.769 ± 7.018	31.531 ± 9.797			

Data are presented as mean ± SD; “*” indicates a significant time main effect (p < 0.05)

(Max: maximum joint angles; Min: minimum joint angles; ROM: range of motion;

RN_time: running time; RN_nonmask: running without mask; RN_mask: running with mask.)

**Table 2:** Kinetic variables of the average PVGRF during treadmill running between the mask and nonmask groups

Characteristic	RN_Time	RN_nonmask	RN_mask	P values		
				Main effect (Group)	Main effect (Times)	Interaction (Group × Times)
Average PVGRF (BW)	0–1 min	2.067 ± 0.250	2.119 ± 0.152	0.707	0.000*	0.697
	5–6 min	2.125 ± 0.254	2.136 ± 0.206			
	9–10 min	2.170 ± 0.228	2.198 ± 0.174			
	14–15 min	2.221 ± 0.258	2.231 ± 0.190			

Data are presented as mean ± SD; “*” indicates a significant time main effect (p < 0.05)

(PVGRF: peak vertical ground reaction forces; RN_time: running time; RN_nonmask: running without mask; RN_mask: running with mask.)

### Kinematics analysis

**Fig. 2:**
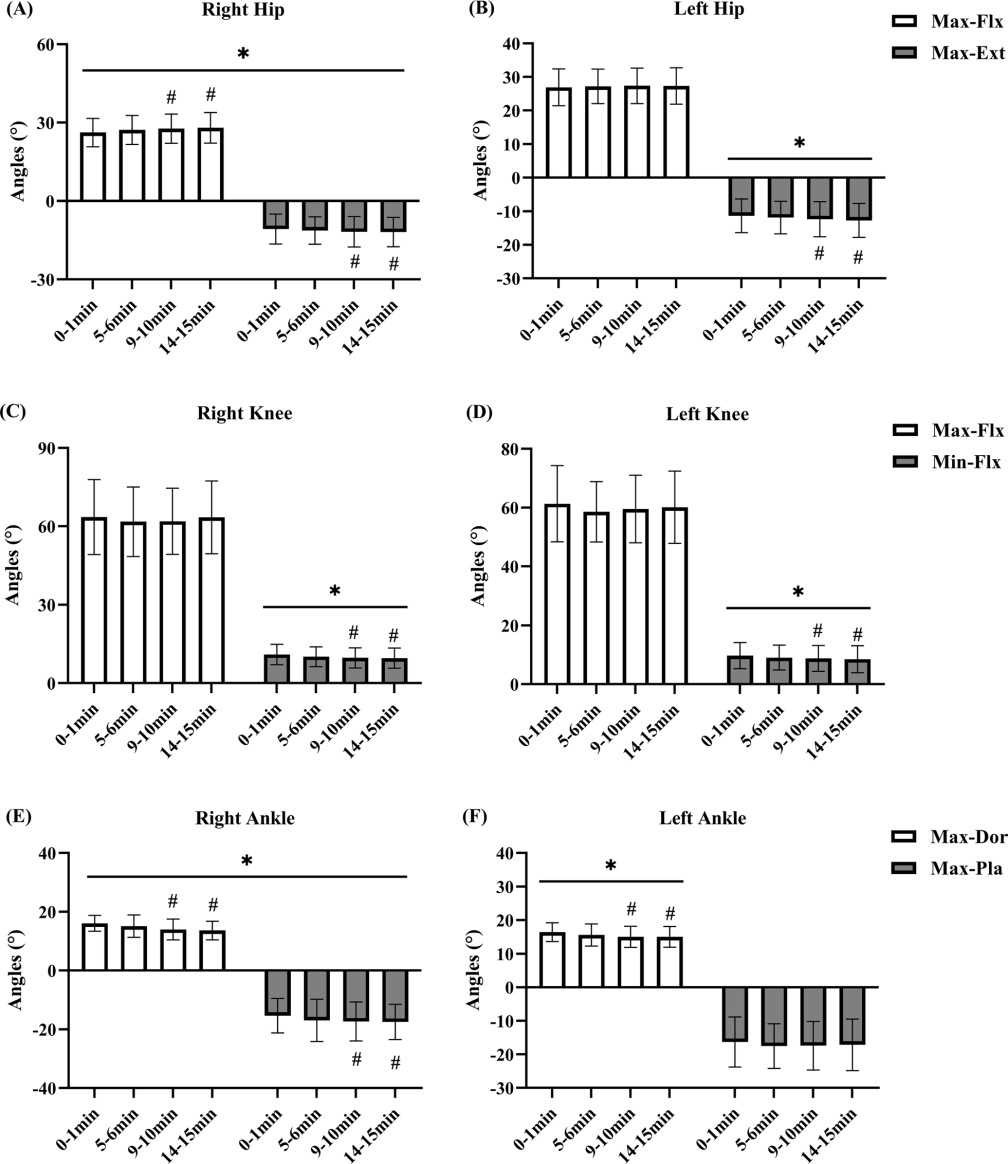
Mean (SD) hip, knee, and ankle joint angle changes for treadmill running over time. “*” Statistically significant main effect of joint angles from 0–1 min to 14–15 min (P < 0.05). “#” Indicates a significant difference from 0–1 min (P < 0.05). Average Maximum Flexion (Max-Flx); Average Maximum Extension (Max-Ext); Average Minimum Flexion (Min-Flx); Average Maximum Dorsiflexion (Max-Dor); Average Maximum Plantarflexion (Max-Pla)

Running time revealed significant main effects on the hip (right: p = 0.002), knees (right: p = 0.001; left: p = 0.001), and ankles (right: p = 0.002; left: p = 0.002) maximum joint angles located in the vertical position anterior to the sagittal plane. Follow-up post hoc comparisons to analyze the differences between times showed that the right hip average flexion max (Fig. 2A) was significantly increased at RN_9–10 min_ and RN_14–15 min_ compared to RN_0–1 min_ (p = 0.020 and p = 0.008, respectively, ES varying from 0.27 to 0.33). The right knee average flexion min (Fig. 2C) was significantly decreased at RN_9–10 min_ and RN_14–15 min_ compared to RN_0–1 min_ (p = 0.012 and p = 0.017, respectively, ES varying from 0.33 to 0.36). The left knee average flexion min (Fig. 2D) was significantly decreased at RN_9–10 min_ and RN_14–15 min_ compared to RN_0–1 min_ (p = 0.039 and p = 0.010, respectively, ES varying from 0.21 to 0.27). The right ankle average dorsiflexion max (Fig. 2E) was significantly decreased at RN_9–10 min_ and RN_14–15 min_ compared to RN_0–1 min_ (p = 0.030 and p = 0.004, respectively, ES varying from 0.66 to 0.82). The left ankle average dorsiflexion max (Fig. 2F) was significantly decreased at RN_9–10 min_ and RN_14–15 min_ compared to RN_0–1 min_ (p = 0.024 and p = 0.016, respectively, ES varying from 0.47 to 0.48). Therefore, the maximum joint angles located in the vertical position anteriorly changed with time, the hip joint flexion angle increased, and the knee joint flexion angle and ankle joint dorsiflexion angle decreased after nine minutes of running.

Running time revealed a significant main effect on the hips (right: p = 0.008, left: p = 0.009) and ankle (right: p = 0.004) maximum joint angles located in the vertical position posterior to the sagittal plane. Follow-up post hoc comparisons to analyze the differences between times showed that the right hip average extension max (Fig. 2B) was significantly increased at RN_9–10 min_ and RN_14–15 min_ compared to RN_0–1 min_ (p = 0.004 and p = 0.001, respectively, ES varying from 0.18 to 0.20). The left hip average extension max (Fig. 2B) was significantly increased at RN_9–10 min_ and RN_14–15 min_ compared to RN_0–1 min_ (p = 0.024, p = 0.038, respectively, ES varying from 0.21 to 0.28). The right ankle average plantar flexion max (Fig. 2E) was significantly increased at RN_9–10 min_ and RN_14–15 min_ compared to RN_0–1 min_ (p = 0.029; p = 0.004, respectively, ES varying from 0.32 to 0.36). Therefore, the maximum joint angles located in the vertical position posteriorly changed with time, the hip joint extension angle increased, and the ankle joint plantar flexion angle increased after nine minutes of running.

**Fig. 3:**
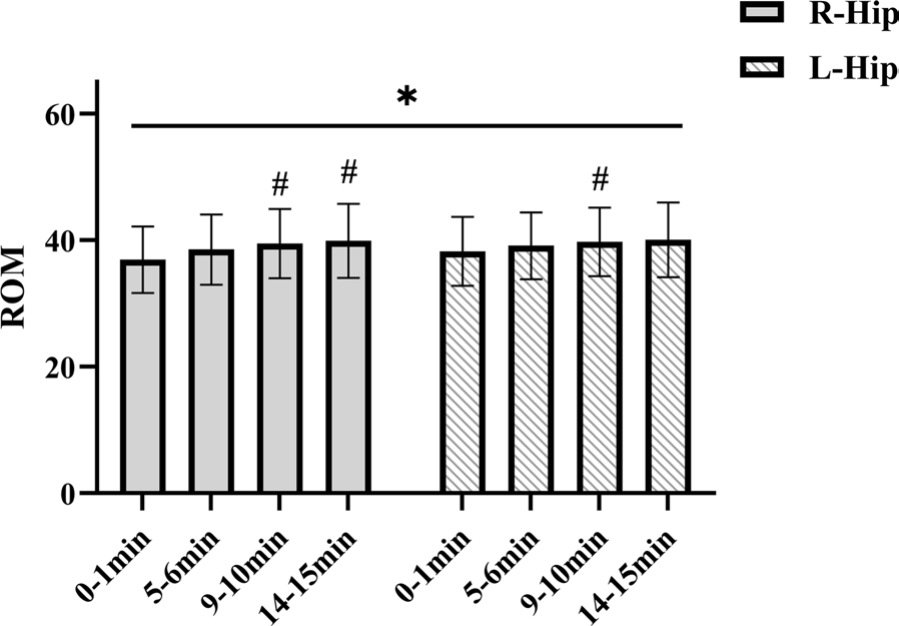
Mean (SD) hip joint range of motion (ROM) changes for treadmill running over time. “*” Statistically significant main effect of ROM from 0–1 min to 14–15 min (P < 0.05). “#” Indicates a significant difference from 0–1 min (P < 0.05)

Running time revealed a significant main effect (Fig. 3) on the hips (right: p = 0.000, left: p = 0.009) joint ROM in the sagittal plane. Follow-up post hoc comparisons to analyze the differences between times showed that the right hip average ROM was significantly increased at RN_9–10 min_ and RN_14–15 min_ compared to RN_0–1 min_ (p < 0.001; p < 0.001, respectively, ES varying from 0.47 to 0.53), and the left hip average ROM was significantly increased at RN_9–10 min_ compared to RN_0–1 min_ (p = 0.029, ES varying from 0.28). Therefore, the hip joint ROM changed with time and increased after nine minutes of running.

### Kinetics analysis

**Fig. 4:**
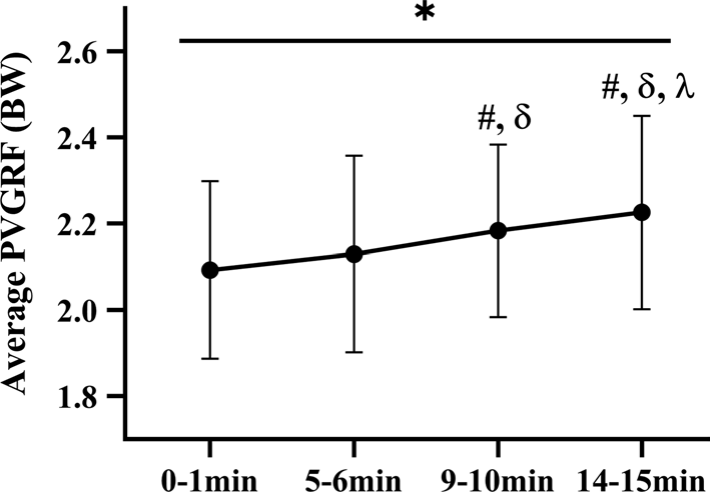
Mean (SD) of average PVGRF changes for treadmill running over time. “*” Statistically significant main effect of average PVGRF from 0–1 min to 14–15 min (P < 0.05). “#” Indicates a significant difference from 0–1 min (P < 0.05). “δ” Indicates a significant difference from 5–6 min (P < 0.05). “λ” indicates a significant difference from 9–10 min (P < 0.05)

Running time revealed a significant main effect (Fig. 4) on the average PVGRF (p < 0.001). The post hoc comparisons showed that running at RN_9–10 min_ and RN_14–15 min_ were significantly increased than running at RN_0–1 min_ (p < 0.001; p < 0.001, respectively, ES varying from 0.45 to 0.62), running at RN_9–10 min_ and RN_14–15 min_ were significantly increased than running at RN_5–6 min_ (p = 0.001; p = 0.001, respectively, ES varying from 0.25 to 0.43), running at RN_14–15 min_ were significantly increased than running at RN_9–10 min_ (p = 0.030, ES = 0.20). Therefore, the average PVGRF of the lower extremities changed with time and increased after nine minutes of running.

## Discussion

The purpose of this study was to investigate whether the sports performance of lower extremities during fifteen min-treadmill running at a 75% VO_2_ max speed was affected by wearing a mask and running time. The kinematics and kinetics results showed that there was no difference between the mask group and the nonmask group during running, and wearing a mask did not affect the joint angle or impact peaks of the lower extremities. As running time increased, hip joint flexion/extension max angles, hip joint ROM, and ankle joint plantar flexion max angles increased after nine min of running; knee joint flexion min angles and ankle joint dorsiflexion max angles decreased after nine min of running; average PVGRF increased after nine min of running. Therefore, the joint angles of the lower extremity and touchdown PVGRF were affected by exercise time and changed after nine min of treadmill running for the mask group and nonmask group.

### Changes in joint angles located in the vertical position anteriorly

The study found that joint angles located vertical position anteriorly were not different between mask group and nonmask group during fifteen min-treadmill running, the joint angles of right hip average flexion max increased, right/left knee average flexion min and right/left ankle average dorsiflexion max decreased with time after nine min-running. The running gait changes adaptively under the influence of repetitive loading of lower extremities. A previous study found that runners tend to maintain the same leg stiffness as running time increases, while knee and hip more extended postures are beneficial for strengthening leg stiffness when the lower extremity touchdown [19]. In this study, the right hip average flexion max increased and the knee average flexion min decreased after nine minutes of running, which may help the runners maintain the same leg stiffness during fifteen minutes of running to adapt to the constant running speed of the treadmill. In addition, a previous study found that runners who increased the dorsiflexion angle of their right ankle by approximately 5° at any time with mechanical perturbation could still adjust their running patterns and maintain stability with a time-dependent adaptive strategy [20]. In this study, the right/left ankle average dorsiflexion max increased after nine minutes of running time, which may be the safety strategy adopted by the runners to maintain the constant speed given by the treadmill.

### Changes in joint angles located in the vertical position posteriorly

The study found that joint angles located vertical position posteriorly were not different between the mask group and nonmask group during fifteen min-treadmill running, but the joint angles of right/left hip average extension max and right ankle average plantarflexion max increased with time after nine minutes running. A previous study found that knee flexion–extension muscle fatigue led to greater hip extension in the toe-off phase of running, while ankle flexion–extension muscle fatigue led to greater ankle plantar flexion in the swing phase of running [21]. In this study, the right/left hip average extension max and right ankle average plantar flexion max increased after nine minutes of running, which may be due to the increased plantar flexion angle for the running propulsive phase of stance with exercise time. In addition, past studies have found that an increased ankle plantar flexion angle during jogging can increase the push-off power of the lower extremities for forward propulsion [22]. Ankle plantar flexor stretch–shortening activity during running can store energy attached to the Achilles tendon for release early and late in the stance phase [23]. In this study, the right ankle average plantar flexion max angle of runners increased after nine minutes of running to maintain a constant running speed, which may help to enhance the elastic energy reserve and increase the push-off power of lower extremities, thereby increasing the body power for forward propulsion.

### Changes in average ROM

The study found that the average ROM was not different between the mask group and the nonmask group during fifteen min-treadmill running, and the average right/left hip ROM increased with time after nine minutes of running. A previous study found that the runner hip, knee, and ankle joint ROM in the sagittal plane during the stance phase increased with fatigue at a fixed speed. The runner maintained the balance of mechanical torque and angular displacement to produce the same level of mechanical power during the running period, while the reduction in joint torque or muscle force caused by the increase in exercise time induced an increase in joint ROM [24]. Therefore, the right/left hip average ROM increased over time, which may be due to a stable strategy adopted by the runner throughout the exercise time to maintain a constant running speed in the entire exercise time. In addition, muscle tuning maintains skeletal activity on the preferred movement path, and the muscle adjusts slightly to adapt to the conditions when it is necessary to maintain the same movement conditions for a period of time [25]. In this study, the average right/left hip ROM increased with time after nine minutes of running, which may be the result of the change in the lower extremity joint angle after the adjustment of muscle activation.

### Changes in the average PVGRF

The study found that the average PVGRF was not different between the mask group and the nonmask group during fifteen min-treadmill running, and the average PVGRF increased with time after nine minutes of running. In the process of running, the VGRF applied to a foot plantar surface would directly affect the load transmitted of each joint for lower extremities with musculoskeletal injuries [26]. The increase in GRF during running increases the metabolic consumption of the body to meet the output of muscle mechanical power, which accumulates the risk of injury from overuse of lower extremity joints [27]. The runners in this study maintained a constant running speed, which may lead to greater vertical ground reaction force and increase the risk of lower extremity joint injury. In addition, a past study found that running fatigue alters the lower-extremity movement patterns of novice runners, making them less prone to knee flexion on landing and more upright on the ground, which may increase the risk of lower-extremity injury [28]. In this study, the decrease in right/left knee average flexion min after nine minutes of running, that is, the lower extremities landing in a more upright manner, may reduce the ability of the knee joint to absorb shock and increase the average PVGRF. An increase in the ankle dorsiflexion angle will directly affect the ability of the lower extremities to absorb impact force and increase GRF [29]. In this study, the right/left ankle average dorsiflexion max decreased after nine minutes of running, which may alleviate the greater stress in the knee joint and control the GRF within a reasonable range to avoid the risk of lower extremity injury.

### Limitations

This study has the following limitations. First, the participants of this study were male college students aged 19–21 years, so the conclusions may not be fully applicable to women of the same age or other age groups. Second, this study focused on the effect of wearing masks and running time on the joint angle and PVGRF of the lower limbs in the postpandemic era, but EMG data were not collected to observe the muscle response of the lower limbs. It is also necessary to further explore muscle activation during treadmill running to make the study more detailed.

### Conclusions

Currently, the global pandemic prevention regulations are constantly updated and changed under the evolution of the new variant of COVID-19, but wearing protective masks in public will still be an important means to block the pandemic virus in the future. In this study, wearing a medical mask during 15 min of treadmill running at a speed of 75% VO_2_ max did not affect the sports performance of the lower extremities, but the joint angles and PVGRF of the lower extremities changed after nine min of running time. Adaptive changes in hip, knee, and ankle joint angles located in the vertical position anteriorly/posteriorly as running time increased between the mask and nonmask groups showed that the runners coped with the fixed speed of the treadmill by changing the joint angles of the lower extremities. At the same time, PVGRF also increased after a nine min running time and may be accompanied increased risk of lower extremity injury. Future research will further explore the impact of long-term exercise with medical masks on human blood biochemical values and possible safety issues in the postpandemic era.

## Data Availability

The data and materials used to support the findings of this study are available from the corresponding author upon request, and the datasets used and analyzed in the current study are included in this article.

## Abbreviations

BMI: Body mass index
PVGRF: Peak vertical ground reaction forces
VO_2_ max: Maximal aerobic capacity
ROM: Ranges of motion
